# A 1-Year Weight Management Program for Difficult-to-Treat Asthma With Obesity: A Randomized Controlled Study

**DOI:** 10.1016/j.chest.2024.09.042

**Published:** 2024-10-18

**Authors:** Varun Sharma, Helen Clare Ricketts, Louise McCombie, Naomi Brosnahan, Luisa Crawford, Lesley Slaughter, Anna Goodfellow, Femke Steffensen, Rekha Chaudhuri, Michael E.J. Lean, Douglas C. Cowan

**Affiliations:** a School of Infection and Immunity, College of Medical, Veterinary & Life Sciences, University of Glasgow, Glasgow, United Kingdom; b Human Nutrition, School of Medicine, Dentistry and Nursing, College of Medical, Veterinary & Life Sciences, University of Glasgow, Glasgow, United Kingdom; c Glasgow Royal, Clinical Research Facility, Glasgow, United Kingdom; d Counterweight Ltd, London, United Kingdom

**Keywords:** asthma, asthma remission, difficult-to-treat asthma, obesity, severe asthma, weight management

## Abstract

**Background:**

Obesity-associated asthma results in increased morbidity and mortality. We report 1-year asthma outcomes with a weight management regimen, the Counterweight-Plus Programme (CWP), compared with usual care (UC) in a single-center, randomized controlled trial in patients with difficult-to-treat asthma and obesity.

**Research Question:**

Can use of the CWP result in improved asthma control and quality of life compared with UC at 1 year in patients with difficult-to-treat asthma and obesity?

**Study Design and Methods:**

Adults with difficult-to-treat asthma and BMI ≥ 30 kg/m^2^ were randomized (1:1 CWP:UC) to treatment. The CWP, with dietitian support, included a 12-week total diet replacement phase (850 kcal/d low-energy formula), and then subsequent food reintroduction and maintenance phases up to 1 year. Outcomes include results of the six-item Asthma Control Questionnaire (ACQ-6) and Asthma Quality of Life Questionnaire (AQLQ), as well as health care usage. A minimal clinically important difference (MCID) is 0.5 for ACQ-6 and AQLQ.

**Results:**

Of 36 patients recruited, 29 attended visits at 52 weeks (13 CWP and 16 UC). The CWP resulted in greater weight change (median, –14 kg [interquartile range (IQR), –15 to –9 kg]) compared with UC (median, 2 kg [IQR, –7 to 8 kg]; *P* = .015) at 52 weeks. A greater proportion achieved MCID with the CWP vs UC in AQLQ (71% vs 6%, respectively; *P* < .001). No between-group differences were observed in ACQ-6. Median exacerbation frequency was reduced over 52 weeks with the CWP from 4 (IQR, 2 to 5) to 0 (IQR, 0 to 2) (*P* < .001), although no between-group difference was observed. Seventy percent of the CWP group lost ≥ 10% body weight and had improvement in ACQ-6 (mean difference, –1.1; 95% CI, –1.9 to –0.3; *P* = .018) and AQLQ (mean difference, 1.2; 95% CI, 0.4, 2.1; *P* = .011) across 52 weeks.

**Interpretation:**

In this study, the use of a dietitian-supported weight management program resulted in sustained weight loss and is a potential treatment for obesity in asthma. The CWP resulted in a higher proportion achieving MCID improvements in AQLQ compared with UC. Within-group differences in AQLQ and exacerbation frequency suggest potential with the CWP. These encouraging signals justify a larger sample study to further assess asthma-related outcomes.

## Graphical Abstract

**Figure:**
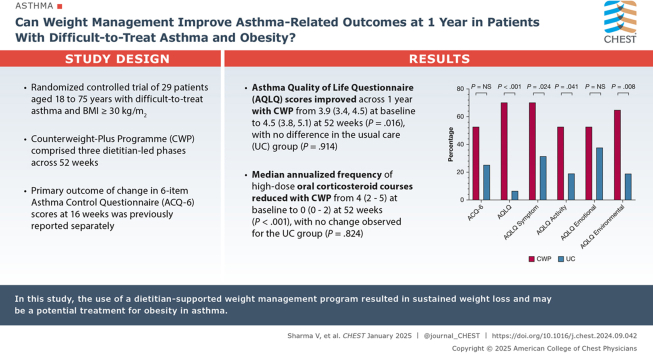

FOR EDITORIAL COMMENT, SEE PAGE 1

**Take-home Points**

**Study Question:** Can use of the Counterweight-Plus Programme (CWP) for weight management improve asthma-related outcomes at 1 year in patients with difficult-to-treat asthma and obesity, compared with those receiving usual care?

**Results:** At 1 year, CWP resulted in a sustained and significant percent weight loss of 12.8% compared with usual care, with a higher proportion of patients experiencing improvement in quality of life compared with usual care. Within-group improvements in asthma exacerbation frequency and asthma-related quality of life were observed with CWP.

**Interpretation:** CWP resulted in persistent and clinically relevant weight loss at 1 year. Although no between-group differences were observed in most asthma-related outcomes, there are compelling signals suggesting value of CWP in the management of the treatable trait obesity in difficult-to-treat asthma.

Obesity is common in uncontrolled asthma,^1^ and sustained weight loss is a challenge in this phenotype that is prone to frequent exacerbations, high treatment burden, and steroid use. Moreover, obesity reduces the chances of achieving clinical asthma remission when receiving biologic therapy.^2^ Asthma remission has become a potentially achievable target, although consensus regarding an appropriate definition has not been reached. One suggested definition by McDowell et al^2^ includes an Asthma Control Questionnaire (ACQ) score < 1.5, no maintenance oral corticosteroid (OCS) or high-dose OCS use for disease control, and an FEV_1_ above the lower limit of normal or < 100 mL from baseline prebiologic initiation. The poor remission rate in the population with severe asthma and obesity further highlights the need to tackle this potentially treatable trait.

Previously, we reported improvements, compared with usual care (UC), in asthma control and quality of life in individuals with difficult-to-treat asthma and obesity over 16 weeks with the Counterweight-Plus Programme (CWP), a weight management regimen, resulting in weight loss of 12 kg.^3^ The current question is whether asthma-related benefits from weight loss are sustained over a longer period.

One-year results from the Diabetes Remission Clinical Trial (DiRECT) in type 2 diabetes mellitus, reported by Lean et al in 2018,^4^ reported a mean weight loss of around 10 kg (*P* < .001) with CWP compared with UC at 12 months. Sustained weight loss and persistent improvements in asthma-related outcomes must be confirmed prior to considering weight management as a therapy for difficult-to-treat asthma with obesity. The current article reports 1-year findings from a randomized controlled feasibility trial of CWP compared with UC in individuals with difficult-to-treat asthma and obesity.

## Study Design and Methods

We conducted a single-center randomized controlled open-label parallel study of a weight management program (CWP) compared with UC (randomized 1:1) in adult patients with difficult-to-treat asthma and obesity. Further details of the population, recruitment, and eligibility have been described previously^3^ but are briefly summarized in the following sections. Participants attended study visits at baseline (visit 1), 16 weeks (visit 2) and 52 weeks (visit 3).

The trial was approved by the West of Scotland Regional Ethics Committee (18/WS/0216) and registered at ClinicalTrials.gov (NCT03858608), where the trial protocol is also described.^5^ Due to the COVID-19 pandemic, face-to-face follow-up visits were replaced with telephone consultations when necessary to optimize data collection.

### Participants

Briefly, adults aged 18 to 75 years with a BMI ≥ 30.0 kg/m^2^ and a diagnosis of difficult-to-treat asthma as per Scottish Intercollegiate Guidelines Network/British Thoracic Society and Global Initiative for Asthma guidelines^6^^,^^7^ were recruited from specialist asthma clinics and ward admissions across NHS Greater Glasgow and Clyde between August 2019 and August 2021. All participants continued standard asthma care with ongoing review at their parent asthma clinics.

### Measurements

Demographic characteristics, anthropomorphic measures, asthma and other medical history details, medication usage, and health care usage were collected at baseline and are as previously described. At all visits, assessments included questionnaires (six-item ACQ [ACQ-6]; Asthma Quality of Life Questionnaire [AQLQ]; Medical Research Council Dyspnea Scale score; Hospital Anxiety Depression scale), venesection, spirometry (ALPHA Spirometer; Vitalograph, Buckingham, UK) as per European Respiratory Society/American Thoracic Society standards,^8^ peak expiratory flow rate, fractional exhaled nitric oxide (Feno) (NIOX VERO, Aerocrine AB; Solna, Sweden) as per American Thoracic Society guidelines,^9^ 6-min walk test as per European Respiratory Society/American Thoracic Society standards,^10^ and accelerometery. Both the ACQ-6 and AQLQ are validated tools assessing control and quality of life, respectively, in asthma.^11^^,^^12^ The minimal clinically important difference (MCID) is 0.5 for both, with an ACQ-6 score ≥ 1.5 consistent with poor asthma control and a higher AQLQ score indicating better quality of life.

### Counterweight-Plus Programme

Details of the CWP protocol have been described previously.^3^ Briefly, the program comprises three dietitian-led phases across 52 weeks. A total diet replacement phase consisting of a low-energy liquid formula (around 850 kcal/d) for 12 weeks for weight loss induction was followed by a food reintroduction phase of stepwise calorie-controlled meals with reducing formula use for 6 weeks. Finally, an approximate 34-week weight maintenance phase of tailored calorie-controlled meals with dietitian review (monthly but with flexibility in frequency and duration of appointments, guided by individual responses and circumstances) completed the program.

### Asthma-Related Outcomes

The primary outcome was the difference in change in ACQ-6 scores from baseline (visit 1) to 16 weeks (visit 2) between CWP and UC as reported previously.^3^ Further asthma-related outcomes included the change in ACQ-6 and AQLQ (including each AQLQ domain) from baseline to 52 weeks and difference in proportion of participants with a change in MCID (≥ 0.5) in the ACQ-6 and AQLQ between CWP and UC at 52 weeks.

### Statistical Analysis

Participants attending visits at baseline (visit 1) and at 52 weeks (visit 3) were included for the intention-to-treat analysis. Continuous data with parametric and nonparametric distribution (assessed using histograms, skewness, kurtosis, and Shapiro-Wilk testing) were described as mean (95% CI) or median (interquartile range [IQR]), respectively, and compared by using unpaired *t* tests or *U* tests, respectively. Comparison across the three time points (visit 1, visit 2, and visit 3) for UC and CWP was performed by using repeated measures analysis of variance or the Friedman test depending on data distribution. Categorical variables are described as number (percentage) and were compared by using the χ^2^ or Fisher exact test as appropriate.

Missing data were analyzed, and key variables missing at random were subjected to multiple imputations.^13^ Missing data for the key asthma-related outcomes ACQ-6, AQLQ, and annualized health care use were missing at random, and a multiple imputation model was used with Rubin’s rules to generate pooled estimates. Five imputations were performed applying an automated method using SPSS with either an iterative Markov chain Monte Carlo or monotone method depending on the pattern of missing values. Other variables with missing data were deemed missing completely at random due to the effect of virtual follow-up visits, and complete case analysis was appropriate here.

The primary outcome time-point was 16 weeks, and sample size was calculated to reflect this. To show a difference of 0.5 (MCID) between mean changes in ACQ-6 in the CWP and UC groups from baseline to 16 weeks, based on an SD of 0.5, a sample size of 30 (15 per group) was required (alpha 0.05, beta 0.2, and power 0.8). A target of 40 was chosen to allow for a 25% dropout rate; however, initial higher-than-expected retention resulted in terminating recruitment early at N = 36.

All analyses of anonymized data were performed by using IBM SPSS Statistics for Mac, version 28 (IBM SPSS Statistics, IBM Corporation), and graphs were produced by using GraphPad Prism for Mac, version 9.5.1 (GraphPad Software). Significance was set at *P* ≤ .05. Post hoc tests are described in each of the following relevant sections.

## Results

**Figure 1:**
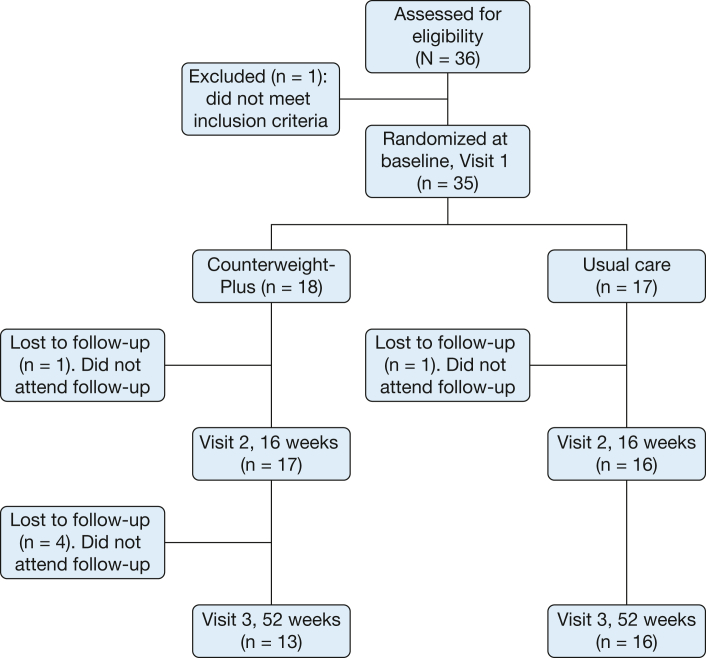
Consolidated Standards of Reporting Trials flowchart.

Baseline characteristics (Table 1) for the CWP and UC groups, described previously,^3^ showed a population with poorly controlled disease and reduced quality of life, high biologic and maintenance OCS use requirements (33% and 17%, respectively), and frequent exacerbations. We recruited 36 participants (one was excluded at baseline due to ineligibility). A total of 33 participants attended visits at 16 weeks (primary outcome); 29 of these participants attended visits at 52 weeks (13 CWP and 16 UC) (Fig 1). Six participants did not attend visits at 1 year: four were lost to follow-up and did not respond to attempts at contact, one was due to mental health issues predating the trial, and one felt unable to commit to the intervention. Baseline characteristics for those attending visits at 52 weeks were similar to those of individuals who did not attend their visits (e-Table 1). Multiple imputation was applied for certain missing variables as described in the Study Design and Methods section; complete case analysis is reported in e-Table 2. Comparison of change in weight, ACQ-6, and AQLQ from baseline (visit 1) to 16 weeks (visit 2) between those who attended visits at 52 weeks and those who did not in the intervention group showed a significant improvement in weight and AQLQ in those who attended visits at 52 weeks and no improvement in those who did not (e-Table 3).

**Table 1:** Baseline Characteristics

Variable	Overall (N = 35)	CWP (n = 18)	UC (n = 17)
Age, y	52.6 (48.3, 56.9)	56.7 (51.3, 62.1)	48.3 (41.5, 55.1)
Female sex	22 (62.9)	13 (72.2)	9 (52.9)
Smoking status			
Currently smokes	1 (2.9)	0 (0.0)	1 (5.9)
Formerly smoked	19 (54.3)	12 (66.7)	7 (41.2)
Never smoked	15 (42.9)	6 (33.3)	9 (52.9)
Smoking (pack-y)	15.0 (6.0-30.0)	15.0 (5.0-22.5)	5.0 (0.0-20.0)
Age at asthma diagnosis, y	30.9 (23.8, 38.1)	34.3 (24.1, 44.4)	27.4 (16.6, 38.2)
Duration of asthma, y	21.7 (16.5, 27.0)	22.5 (13.7, 31.3)	20.9 (14.3, 27.5)
Atopy	25 (71.4)	12 (66.7)	13 (76.5)
Allergic rhinitis	19 (54.3)	9 (50.0)	10 (58.8)
Perennial rhinitis	16 (45.7)	7 (38.9)	9 (52.9)
Nasal polyps	4 (11.4)	3 (16.7)	1 (5.9)
Nasal surgery	4 (11.4)	3 (16.7)	1 (5.9)
Eczema	13 (37.1)	6 (33.3)	7 (41.2)
GORD	30 (85.7)	16 (88.9)	14 (82.4)
ILO/DFB	8 (22.9)	5 (27.8)	3 (17.6)
Psychological illness	18 (51.4)	8 (44.4)	10 (58.8)
Emphysema	5 (14.3)	3 (16.7)	2 (11.8)
Bronchiectasis	1 (2.9)	1 (5.6)	0 (0.0)
SAFS/ABPA	9 (25.7)	3 (16.7)	6 (35.3)
Diabetes mellitus	4 (11.4)	4 (22.2)	0 (0.0)
Hypertension	9 (25.7)	6 (33.3)	3 (17.6)
Cardiac disease	7 (20.0)	2 (11.1)	5 (29.4)
Osteopenia/osteoporosis	15 (42.9)	6 (33.3)	9 (52.9)
ICS/LABA	35 (100.0)	18 (100.0)	17 (100.0)
BDP equivalent dose, μg	1,600 (1,600-2,000)	1,600 (1,600-1,600)	2,000 (1,600-2,400)
LAMA	33 (94.3)	18 (100.0)	15 (88.2)
Maintenance prednisolone	6 (17.1)	4 (22.2)	2 (11.8)
Prednisolone dose, mg	4.5 (1.2, 7.8)	4.5 (–1.9, 10.9)	4.5 (–1.9, 10.9)
Montelukast	27 (77.1)	14 (77.8)	13 (76.5)
Theophylline	22 (62.9)	10 (55.6)	12 (70.6)
Azithromycin	7 (20.0)	6 (33.3)	1 (5.9)
Omalizumab	4 (11.4)	1 (5.6)	3 (17.6)
Mepolizumab	8 (22.9)	4 (22.2)	4 (23.5)
Antihistamine	24 (68.6)	11 (61.1)	13 (76.5)
Nasal steroid	24 (68.6)	12 (66.7)	12 (70.6)
PPI/H2A	30 (85.7)	17 (94.4)	13 (76.5)
Previous 12 mo			
Prednisolone courses	3 (2-5)	4 (2-5)	3 (2-5)
Out-of-hours GP visit	0 (0-0)	0 (0-0)	0 (0-0)
ED visit	0 (0-0)	0 (0-0)	0 (0-0)
Hospital admissions	0 (0-1)	0 (0-0)	0 (0-1)
ICU admissions	0 (0-0)	0 (0-0)	0 (0-0)
Weight, kg	101.7 (91.4-118.7)	103.3 (96.9-118.3)	97.0 (86.5-122.0)
BMI, kg/m^2^	37.5 (35.0-42.3)	38.2 (35.6-45.3)	36.1 (32.7-42.5)
MRC Dyspnea Scale	3 (3-4)	3 (3-4)	3 (3-4)
ACQ-6 score	2.8 (2.4, 3.1)	2.8 (2.2, 3.3)	2.7 (2.2, 3.3)
AQLQ score			
Overall	3.8 (3.4, 4.2)	3.8 (3.3, 4.4)	3.8 (3.2, 4.4)
Symptom domain	3.8 (3.4, 4.2)	3.7 (3.2, 4.3)	3.8 (3.2, 4.5)
Activity domain	3.8 (3.4, 4.2)	3.9 (3.4, 4.4)	3.7 (3.0, 4.3)
Emotional domain	3.8 (3.2, 4.3)	3.6 (2.8, 4.5)	3.9 (3.1, 4.7)
Environmental domain	4.1 (3.6, 4.6)	4.0 (3.4, 4.6)	4.2 (3.4, 5.0)
HADS			
Anxiety score	8 (6-11)	9 (7-11)	7 (5-11)
Depression score	8 (5-11)	8 (5-11)	9 (7-14)
Eosinophils (×10^9^/L)	0.11 (0.08-0.42)	0.17 (0.08-0.42)	0.1 (0.04-0.51)
Feno, ppb	18 (11-33)	15 (10-35)	20 (13-51)
PEF, L/min	375 (334, 415)	318 (275, 360)	435 (374, 496)
Spirometry			
Pre-BD FEV_1_, %	72.1 (66.0, 78.1)	65.8 (57.1, 74.6)	78.7 (70.7, 86.7)
Pre-BD FEV_1_/FVC, %	70.4 (67.2, 73.5)	67.9 (62.5, 73.2)	73.0 (69.7, 76.2)
Post-BD FEV_1_ change, %	3.4 (1.3, 5.4)	5.1 (1.5, 8.7)	1.5 (–0.5, 3.6)
6MWD, m	326 (284, 367)	315 (250, 381)	337 (282, 393)

Continuous variables are presented as mean (95% CIs) or median (first quartile-third quartile). Categorical variables are presented as No. (%). (Adapted with permission from Sharma et al.^3^) 6MWD = 6-minute walk distance; ABPA = allergic bronchopulmonary aspergillosis; ACQ-6 = six-item Asthma Control Questionnaire; AQLQ = Asthma Quality of Life Questionnaire; BD = bronchodilator; BDP = beclomethasone dipropionate; CWP = Counterweight-Plus Programme; DFB = dysfunctional breathing; Feno = fractional exhaled nitric oxide; GORD = gastro-esophageal reflux disease; GP = general practitioner; HAD = Hospital Anxiety and Depression Scale; H2A = H_2_-receptor antagonist; ICS = inhaled corticosteroid; ILO = inducible laryngeal obstruction; LABA = long-acting beta_2_-agonist; LAMA = long-acting anti-muscarinic antagonist; MRC = Medical Research Council; PEF = peak expiratory flow; ppb = parts per billion; PPI = proton pump inhibitor; SAFS = severe asthma with fungal sensitization; UC = usual care.

### Asthma Outcomes

#### Six-Item Asthma Control Questionnaire

Mean ACQ-6 score was reduced by 0.5 (95% CI, –0.2 to 1.1) from baseline to 52 weeks with CWP and by 0.1 (95% CI, –0.6 to 0.7) with UC, with a mean difference in change in ACQ-6 score between groups of 0.4 (95% CI, –0.5 to 1.2; *P* = .409) (Table 2). Repeated measures analysis of variance indicated no significant changes in ACQ-6 score for CWP (*P* = .168) or UC (*P* = .465) across the three visits.

**Table 2:** Intention-to-Treat Analysis Comparing Asthma-Related and Anthropomorphic Outcomes Over 1 Year Between CWP and UC

Variable	Group	N	Mean (95% CI)/Median (IQR)			Repeated Measures ANOVA/Friedman Test		V1-V3	
			V1	V2	V3	*P* Value	Effect Size	Change in Variable	*P* Value
ACQ-6	CWP	17	2.6 (2.1, 3.2)	2.2 (1.5, 2.8)	2.2 (1.6, 2.8)	.168	0.105	–0.5 (–1.1, 0.2)	
	UC	16	2.7 (2.2, 3.3)	2.9 (2.3, 3.6)	2.6 (2.0, 3.3)	.465	0.050	–0.1 (–0.7, 0.6)	.409
AQLQ	CWP	17	3.9 (3.3, 4.5)	4.7 (4.2, 5.3)	4.5 (3.9, 5.1)	**.016**	0.227	0.6 (–0.1, 1.2)	
	UC	16	3.8 (3.2, 4.5)	3.9 (3.4, 4.5)	3.9 (3.3, 4.6)	.914	0.006	0.1 (–0.5, 0.7)	.254
AQLQ symptom domain	CWP	17	3.8 (3.2, 4.5)	4.8 (4.3, 5.4)	4.5 (3.8, 5.1)	**.010**	0.249	0.6 (–0.1, 1.4)	
	UC	16	3.9 (3.2, 4.6)	4.1 (3.6, 4.7)	4.2 (3.5, 4.9)	.315	0.074	0.4 (–0.3, 1.0)	.595
AQLQ activity domain	CWP	17	4.0 (3.4, 4.5)	4.5 (3.9, 5.1)	4.3 (3.7, 5.0)	.149	0.112	0.4 (–0.3, 1.0)	
	UC	16	3.7 (3.0, 4.3)	3.5 (2.9, 4.2)	3.6 (2.9, 4.3)	.916	0.006	–0.1 (–0.7, 0.6)	.370
AQLQ emotional domain	CWP	17	3.7 (2.9, 4.6)	5.2 (4.4, 5.9)	4.5 (3.8, 5.2)	**.004**	0.289	0.8 (–0.1, 1.7)	
	UC	16	3.9 (3.0, 4.8)	4.6 (3.9, 5.2)	4.3 (3.5, 5.0)	**.039**	0.195	0.3 (–0.2, 0.8)	.357
AQLQ environmental domain	CWP	17	4.1 (3.4, 4.8)	4.6 (3.8, 5.4)	4.8 (3.9, 5.6)	.157	0.109	0.7 (–0.1, 1.4)	
	UC	16	4.2 (3.4, 5.0)	3.7 (2.9, 4.5)	3.7 (2.8, 4.6)	.432	0.054	–0.5 (–1.5, 0.5)	**.047**
Weight, kg^a^	CWP	9	101.7 (95.5 to 112.0)	88.8 (82.0 to 90.7)	87.1 (85.9 to 93.3)	**.001**	0.778	–14.0 (–14.8 to –9.2)^b^	
	UC	8	106.0 (80.9 to 128.0)	105.6 (80.9 to 124.9)	108.6 (87.1 to 145.5)	.417	0.109	1.9 (–7.3 to 7.9)^b^	**.015**
BMI, kg/m^2a^	CWP	9	37.5 (35.6 to 41.8)	32.6 (30.1 to 35.1)	33.1 (31.4 to 37.6)	**.004**	0.613	–4.2 (–6.4, –2.0)	
	UC	8	37.1 (31.5 to 47.8)	37.2 (31.0 to 47.0)	37.5 (32.6 to 54.4)	.417	0.109	–0.1 (–3.6, 3.4)	**.036**
Annualized prednisolone courses^a^	CWP	17	4 (2 to 5)	0 (0 to 5)	0 (0 to 2)	**.001**	0.435	–3 (–5, –1)	
	UC	16	3 (2 to 5)	3 (0 to 6)	2 (1 to 4)	.824	0.012	–1 (–3, 1)	.109

Data are presented as mean (95% CI) and compared with repeated measures analysis of variance (F-statistic and effect size η_p_^2^ [partial eta squared]). Visit 1 (V1) to visit 3 (V3) variables are described as mean (95% CI) and were compared with independent *t* test. Annualized variable compares change from baseline data (number of events in prior 12 months) to 52 weeks ([number of events × 365]/number of days between visits). Boldface highlights significant results (*P* < .05). ACQ-6 = six-item Asthma Control Questionnaire; ANOVA = analysis of variance; AQLQ = Asthma Quality of Life Questionnaire; CWP = Counterweight-Plus Programme; IQR = interquartile range; UC = usual care; V2 = visit 2.

a If data were nonparametric, these variables are described as median (interquartile range) and were compared with Friedman χ^2^ test (effect size Kendall’s W).

b If data were nonparametric, these variables are described as median (interquartile range) and were compared with *U* test.

**Figure 2:**
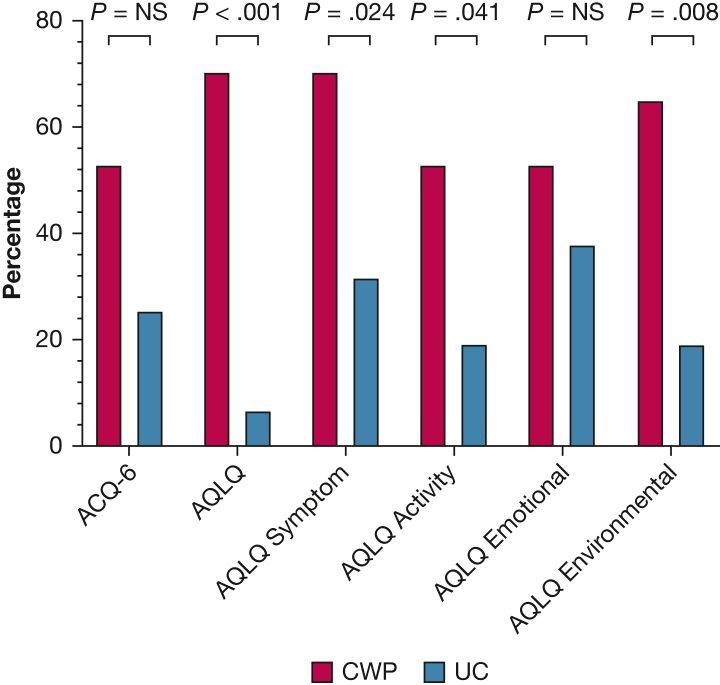
Proportion of participants achieving minimal clinically important difference in ACQ-6 (–0.5) and AQLQ (0.5) scores with the CWP program and UC over 1 year. Compared by using the χ^*2*^ or Fisher exact test. ACQ-6 = six-item Asthma Control Questionnaire; AQLQ = Asthma Quality of Life Questionnaire; CWP = Counterweight-Plus Programme; NS = not significant; UC = usual care.

The proportion of participants achieving MCID in ACQ-6 was greater at 16 weeks with CWP than with UC (53% vs 19%, respectively; *P* = .041) but not at 52 weeks (53% vs 25%; *P* = .101) (Fig 2, Table 3). The 53% of participants achieving MCID at 16 weeks with CWP had sustained this improvement at 52 weeks.

**Table 3:** Proportion of Intention-to-Treat Participants Achieving MCID in Asthma Control and Quality-of-Life Scores at 16 and 52 Weeks

Variable	16 Weeks			52 Weeks		
	CWP Program (n = 17)	UC (n = 16)	*P* Value	CWP Program (n = 17)	UC (n = 16)	*P* Value
ACQ-6	9 (52.9)	3 (18.8)	**.041**	9 (52.9)	4 (25.0)	.101
AQLQ	10 (58.8)	5 (31.3)	.112	12 (70.6)	1 (6.3)	**< .001**
AQLQ symptom domain	12 (70.6)	7 (43.8)	.119	12 (70.6)	5 (31.3)	**.024**
AQLQ activity domain	8 (47.1)	4 (25.0)	.188	9 (52.9)	3 (18.8)	**.041**
AQLQ emotional domain	12 (70.6)	12 (75.0)	1.000	9 (52.9)	6 (37.5)	.373
AQLQ environmental domain	10 (58.8)	5 (31.3)	.112	11 (64.7)	3 (18.8)	**.008**

*P* value compares CWP vs UC using either the χ^2^ or Fisher exact test. Boldface highlights significant results (*P* < .05). ACQ-6 = six-item Asthma Control Questionnaire; AQLQ = Asthma Quality of Life Questionnaire; CWP = Counterweight-Plus Programme; MCID = minimal clinically important difference; UC = usual care.

**Figure 3:**
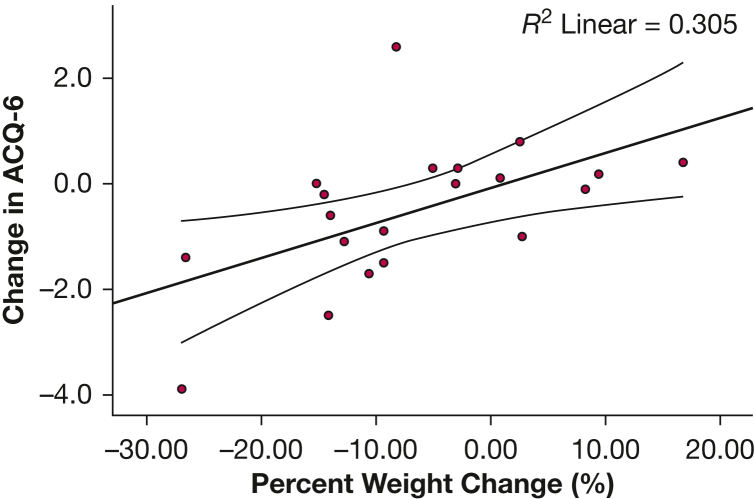
Scatter plot showing correlation between change in ACQ-6 and percent weight change over 1 year with line of best fit and 95% CI. ACQ-6 = six-item Asthma Control Questionnaire.

The Pearson test showed a moderate to large positive correlation between percent change in weight and change in ACQ-6 score across 1 year (*r* = 0.552; *P* = .012). Univariate linear regression was significant (F[1,18] = 7.894; *P* = .012; *R*^2^ = 30.5%), and weight change predicted change in ACQ-6 over 1 year (β = 0.066; 95% CI, 0.017-0.116; *P* = .012) (Fig 3).

#### Asthma Quality of Life Questionnaire

Mean AQLQ score improved across 1 year with CWP from 3.9 (95% CI, 3.4 to 4.5) at baseline to 4.5 (95% CI, 3.8 to 5.1) at 52 weeks (*P* = .016), with no difference in the UC group (*P* = .914) (Table 2). A post hoc pairwise comparison with Bonferroni correction showed improvement in AQLQ with CWP between baseline and 16 weeks (0.8; 95% CI, 0.1-1.5; *P* = .016) and no difference between 16 weeks and 52 weeks (–0.2; 95% CI, –0.9 to 0.4; *P* = 1.000). At 52 weeks, a greater proportion of participants in the CWP group compared with the UC group achieved MCID in AQLQ (71% vs 6%; *P* < 0.001) (Fig 2, Table 3). Individual AQLQ domain results show similar trends (see see supplementary material, section AQLQ domains).

**Figure 4:**
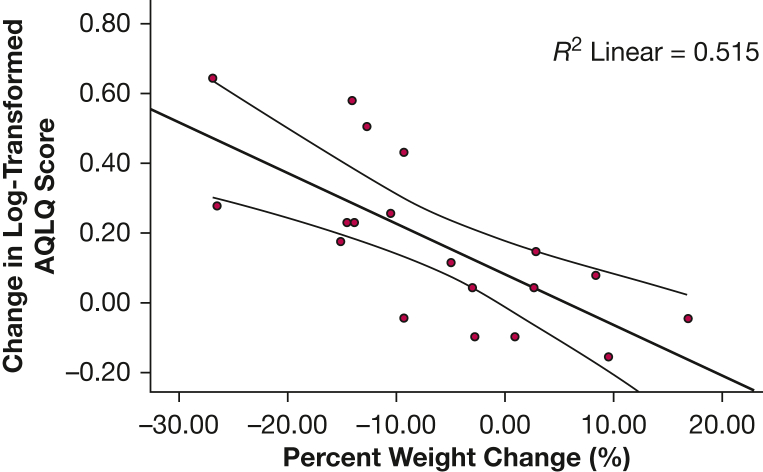
Scatter plot showing correlation between change in log-transformed AQLQ and percentage weight change across 1 year with line of best fit and 95% CI. AQLQ = Asthma Quality of Life Questionnaire.

The Pearson test showed a large negative correlation between percent change in weight and change in log-transformed AQLQ across 52 weeks (*r* = –0.717; *P* < .001). Univariate linear regression was significant (F[1,17] = 18.029; *P* < .001; *R*^2^ = 51.5%), and weight change predicted change in log-transformed AQLQ over 52 weeks (β = –0.015; 95% CI, –0.022 to –0.007; *P* < .001) (Fig 4).

### Other Key Outcomes

Median annualized frequency of high-dose OCS courses (a surrogate for asthma exacerbation) was reduced with CWP from 4 (IQR, 2-5) at baseline to 0 (IQR, 0-2) at 52 weeks (*P* < .001), with no change observed for the UC group (*P* = .824) (Table 2). No between-group differences were observed. The Pearson correlation test showed a moderate positive correlation between change in weight and change in high-dose prednisolone courses (*r* = 0.496; *P* = .026). Univariate linear regression was significant (F[1,18] = 5.9; *P* = .026; *R*^2^ = 25%), and weight change predicted exacerbation frequency over 52 weeks (β = 0.148; 95% CI, 0.020, 0.275; *P* = .026).

**Figure 5:**
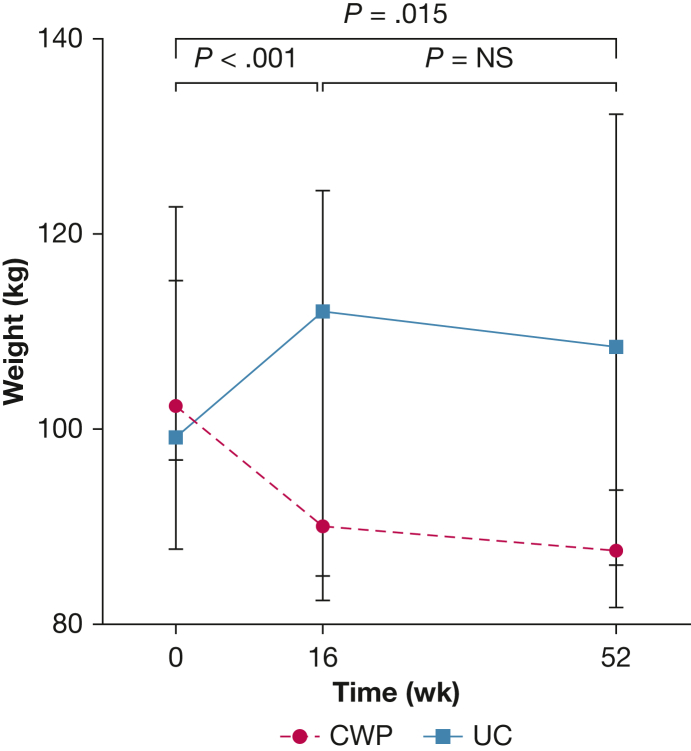
Comparison of median (interquartile range) weight between CWP and UC over 1 year. *P* values compare CWP against UC using the U test. CWP = Counterweight-Plus Programme; NS = not significant; UC = usual care.

Significant weight loss occurred with CWP from a median of 101.7 kg (IQR, 95.5-112.0 kg) at baseline to 87.1 kg (IQR, 85.9-93.3 kg) at 52 weeks (*P* < .001), with no change for UC (*P* = .417) (Table 2). Median weight change was –14 kg (IQR, –14.8 to –9.2 kg) over 52 weeks with CWP and 1.9 kg (IQR, –7.3 to 7.9 kg) with UC (*P* = .015) (Fig 5). Mean percent weight change across 1 year was –12.8% (95% CI, –16.9% to –8.7%) for CWP and 0.0% (95% CI, –8.5% to 8.5%) with UC (mean difference, –12.8%; 95% CI, –21.6 to –4.1%; *P* = 0.007). CWP was associated with similar improvements in BMI as summarized in Table 2.

### Post Hoc Analyses: Asthma Remission, Type 2 Biomarkers, and Weight Loss Comparison

#### Asthma Remission

Using the limited criteria (ACQ-6 score < 1.5, no OCS boosts or maintenance OCS) of asthma remission at 52 weeks, four participants (23.5%) in the CWP group and no participants in the UC group experienced remission (Fisher exact test, *P* = .103). FEV_1_ data were insufficient to be included, although the clinical relevance of this criterion is debated.^2^

### Comparison of CWP Participants Stratified According to Type 2 Status

Post hoc analysis comparing outcomes over 1 year of CWP in individuals stratified by using type 2 biomarkers used paired *t* tests or Wilcoxon signed-rank tests for data with parametric and nonparametric distributions, respectively (e-Table 4). For the purposes of this analysis, type 2 high disease was defined as having Feno ≥ 25 parts per billion and/or blood eosinophil count ≥ 0.15 × 10^9^/L at baseline,^14^ with low levels of both defining type 2 low disease; a *P* value < .1 was accepted to suggest a trend. Trends suggesting improvement were observed in the type 2 high group (n = 11) for ACQ-6 (mean difference, –0.7; 95% CI, –1.5 to 0.1; *P* = .067), AQLQ (mean difference, 0.7; 95% CI, 0.1-1.3; *P* = .036), AQLQ symptom domain (mean difference, 0.8; 95% CI, 0.2-1.4; *P* = .012), AQLQ emotional domain (mean difference, 1.1; 95% CI, 0.3-1.9; *P* = .010), AQLQ environmental domain (mean difference, 0.9; 95% CI, –0.1 to 1.9; *P* = .081), number of OCS courses (median difference, –2, IQR, –6 to 0; *P* = .003), and number of hospital admissions (median difference, 0, IQR, –1 to 0; *P* = .083). No differences were observed in the type 2 low group (n = 6).

### Comparison of CWP Participants Stratified According to Percent Weight Loss

e-Table 5 compares outcomes of CWP in individuals stratified according to weight loss (≥ 10% vs < 10% weight loss) over 52 weeks, again using either paired *t* tests or Wilcoxon signed-rank tests as appropriate. Seventy percent of participants in the CWP group lost ≥ 10% total body weight. Weight loss of 10% was selected following a previously reported suggestion that this was the level required to observe improvement in asthma control and quality of life measures.^3^ Over 52 weeks, improvements were observed in the ≥ 10% weight loss group in ACQ-6 (mean difference, –1.1; 95% CI, –1.9 to –0.3; *P* = .018), AQLQ (mean difference, 1.2; 95% CI, 0.4-2.1; *P* = .011), AQLQ symptom domain (mean difference, 1.3; 95% CI, 0.4-2.2; *P* = .010), AQLQ activity domain (mean difference, 1.0; 95% CI, 0.0 to 2.0; *P* = .052), AQLQ emotional domain (mean difference 1.3; 95% CI, –0.2 to 2.8; *P* = .074), AQLQ environmental domain (mean difference, 1.3; 95% CI, 0.4-2.2; *P* = .011), number of prednisolone courses (mean difference, –4; 95% CI, –7 to –1; *P* = .018), and MRC dyspnea score (mean difference, –1; 95% CI, –2 to –1; *P* = .024). No differences were observed in the < 10% weight loss group.

## Discussion

We report results from a randomized controlled trial assessing asthma-related outcomes at 1 year of use of a weight management program compared with UC. Participants in the CWP group experienced sustained weight loss and reduction in BMI over 1 year compared with UC. Despite this, no between-group differences were observed in ACQ-6 or AQLQ, likely because this study was underpowered at the 1-year time-point. This was a feasibility study powered to assess outcomes at 16 weeks, and as such resulted in a lower sample size at 1 year. Importantly, CWP resulted in a greater proportion of individuals achieving clinically significant (≥ 0.5) improvement in AQLQ compared with UC (71% vs 5%, respectively; *P* < .001). Likewise, CWP was associated with a greater proportion achieving clinically significant improvements in AQLQ symptom, activity, and environmental domains. Correlation and regression analyses support improvements in ACQ-6, AQLQ, and exacerbation frequency with weight loss. This, combined with the encouraging results at 16 weeks, suggests that a larger sample study is warranted. Reassuringly, 53% of the CWP group achieved MCID in ACQ-6 at 16 weeks and, in these individuals, improvements were sustained at 1 year. Moreover, 70% of the CWP group at 1 year lost ≥ 10% weight and showed marked improvement in ACQ-6 compared with baseline, although these numbers are small.

In interpreting our results, we must recognize that weight management is not easy and may not be perceived by all people with obesity and asthma as generating sufficient net benefit to persist. Participants who failed to provide 52-week data were those who showed no improvements in weight, ACQ-6, or AQLQ over the first 16 weeks. The missing data at 52 weeks and the use of multiple imputations may have introduced bias, particularly in small sample sizes. However, the complete case analyses yielded broadly similar results, which supports the interpretation that the benefits are real and provide confidence that a larger study might confirm and quantify significant impacts on asthma control at 1 year. It should be noted, however, that we have not reported data where numbers are extremely low (eg, spirometry data [n = 2]).

Encouragingly, over 1 year, CWP was associated with a reduction in frequency of high-dose OCS courses (a surrogate for asthma exacerbation) while there was no change for UC. However, no between-group differences were observed. Furthermore, this measure was reliant on both participant recall and electronic case record review where possible and was therefore subject to recall bias.

Post hoc analysis revealed that the beneficial effects on asthma control, quality of life, and exacerbation frequency were greater in those losing more weight (≥ 10% vs < 10% weight), although caution must be taken in interpreting these findings due to low group numbers. No between-group difference in “asthma remission” (based on limited available criteria) was observed; however, the prospect of a nonpharmacologic option for asthma remission is appealing, and the 23.5% proportion of remission with CWP may be an optimistic signal upon which to build. Interestingly, participants with type 2-high profiles also seemed to respond more than those with type 2-low disease for ACQ-6, AQLQ, and number of prednisolone courses, although this should also be interpreted with caution. This seems contrary to our understanding of asthma and obesity, which is often associated with type 2-low profiles. To our knowledge, this is the first report comparing effects of weight loss on asthma outcomes in type 2-high and type 2-low asthma. A study by Pinkerton et al^15^ identified a link between type 2-high inflammation and obesity. They observed increased levels of IL-5, IL-13, and CC chemokine receptor type 3 with obesity, the latter involved with eosinophil chemotaxis. Links between adipokine imbalance and eosinophil biology dysfunction have been described previously.^16^, ^17^, ^18^ A comprehensive understanding of the effects of obesity on airway inflammation, and conversely on weight loss, in people with asthma is lacking.

Most studies of weight loss in asthma to date have evaluated short-term outcomes, with few studies documenting long-term benefits. Freitas et al^19^ reported improvements in weight loss, as well as ACQ and AQLQ, after 3 months using an exercise regimen compared with a sham group. At 12 months, weight gain was observed in the intervention group; however, values were not reported, and neither was the effect on ACQ or AQLQ scores. Johnson et al^20^ performed a single-arm study of an online weight loss intervention in participants with obesity and uncontrolled asthma (n = 43) and observed improved asthma control and quality of life in those who lost > 5% body weight. However, as well as lacking a control group, patients were followed up for 6 months in total. Only Ma et al^21^ assessed ACQ at 1 year using a behavioral and lifestyle interventional protocol including calorie restriction compared with UC and found no improvement. However, this population had a lower baseline mean ACQ score (1.4), suggesting a population with better-controlled disease than those in our study, and the weight loss observed was considerably lower (5 kg) than with CWP, likely inadequate to result in improvement in asthma control or quality of life.

The current study has clinical strengths in addressing a difficult-to-treat patient group in a real-life setting. Potential limitations and risk of bias, inevitable in a real-world study, have previously been acknowledged.^3^ These include missing data for laboratory-measured variables such as lung function, blood tests, Feno, 6-min walk test, and accelerometery due to the effects of the COVID-19 pandemic, and for 1 year follow-up patient data sets through loss to follow-up. Results should be considered exploratory given the small sample size and aforementioned limitations. A larger definitive trial is now justified to confirm and quantify the impact of sustained weight loss on treatment demands, exacerbation rates, spirometry, and markers of inflammation, in particular type 2 inflammation. Further study is also warranted to understand the mechanisms involved between weight loss and improved asthma outcomes. Recent approval of antiobesity medications such as glucagon-like peptide-1 receptor agonists^22^ may prove beneficial in the context of asthma.^23^ However, these primarily subcutaneous drugs are not without risk^24^^,^^25^ and may not be attractive options to all patients. Conservative options should be considered for those in whom drug therapies have not been effective. Nonpharmacologic options, such as the CWP, should continue to be explored to provide alternatives to both surgical and pharmacologic options in obesity and asthma.

## Interpretation

To our knowledge, this is the first study of weight management in participants with obesity and asthma to observe sustained weight loss at 1 year and to document benefits in both anthropometric and asthma-related outcomes, specifically asthma-related quality of life. Encouragingly, although no between-group differences were observed at 1 year in asthma control, those who experienced improvement in asthma control at 4 months displayed sustained improvement longer term. Moreover, the majority in the intervention arm who experienced weight loss > 10% had significant asthma control improvement. Within-group improvement in frequency of high-dose OCS courses with CWP also suggests potential of weight loss in reducing asthma exacerbations. The question of asthma remission with CWP remains, with a larger sample size study a potential way to clarify this issue. Finally, we offer insight into a potential effect of weight loss in type 2-high asthma necessitating further attention to obesity-mediated airway inflammation.

## Funding/Support

The trial was sponsored and funded by an NHS Greater Glasgow and Clyde Endowment Fund.

## Financial/Nonfinancial Disclosures

The authors have reported to *CHEST* the following: V. S. and H. C. R. have received travel awards to attend conferences. V. S. has received fees for presentations from AstraZeneca. N. B. has received funding from Cambridge Weight Plan for PhD program and conference and travel expenses and has shares in and is an employee of Counterweight Ltd. R. C. has received funding from AstraZeneca for a study within a Medical Research Council project as investigator lead; has received payment for lectures from GSK, AstraZeneca, Teva, Chiesi, Sanofi, and Novartis; has received funding to attend conferences from Chiesi, Sanofi, and GSK; and has participated on advisory board meetings for GSK, AstraZeneca, Teva, Chiesi, and Novartis. M. E. J. L. has received grants from the National Institute for Health and Care Research, Diabetes UK, All Saints Educational Trust, Novo Nordisk, and MJ Smith Trust for the study; has received consulting fees from Novo Nordisk and Nestle; has received payment for lectures from Oviva, Merck, Sanofi, and Roche; and is a medical advisor for Counterweight Ltd. None declared (L. M., L. C., L. S., A. G., F. S., D. C. C.).

## Supplementary Data

e-Online Data
