# Supplementary material for: A 1-Year Weight Management Program for Difficult-to-Treat Asthma With Obesity: A Randomized Controlled Study
Source: Chest. 2024 Oct 18;167(1):42–53. doi: 10.1016/j.chest.2024.09.042 (PMC11752128; doi:10.1016/j.chest.2024.09.042)
Supplement: e-Online Data [file mmc1.docx]

**Supplementary information**

**Methods**

*Dysfunctional breathing*

Dysfunctional breathing was defined after specialist chest physiotherapy diagnosis following referral for suspected breathing pattern disorder.

*Other outcomes*

Difference in number of high dose prednisolone courses (surrogate for asthma exacerbation requiring oral corticosteroids) between groups over one year.

Comparison of anthropomorphic measures (weight and BMI) between groups over one year.

**Results**

*AQLQ domains*

Similarly to the overall AQLQ score, AQLQ symptom domain improved with CWP from 3.8 (3.3, 4.4) at baseline to 4.5 (3.8, 5.1) at one year (p = 0.010) with no difference for UC (p = 0.315). Pairwise comparison with Bonferroni correction showed improvement in AQLQ symptom domain with CWP between V1 and V2 (1.0 [0.3, 1.7]; p = 0.004) and no difference between V2 and V3 (-0.4 [-1.2, 0.4]; p = 0.674).

There was no significant change in AQLQ activity domain in either group across the three visits.

AQLQ emotional domain improved with CWP from 3.7 (2.8, 4.6) at baseline to 4.5 (3.8, 5.2) at one year (p = 0.004) and with UC from 3.9 (3.1, 4.8) at baseline to 4.3 (3.5, 5.0) at one year (p = 0.039). Pairwise comparison with Bonferroni correction showed improvement in AQLQ emotional domain with CWP between V1 and V2 (1.5 [0.4, 2.6]; p = 0.008) and no difference between V2 and V3 (-0.7 [-1.7, 0.4]; p = 0.318).

No changes were observed in AQLQ environmental domain for CWP (p = 0.157) or UC (p = 0.432) across the three visits, though pairwise comparison with Bonferroni correction suggested a trend towards improvement of 0.7 (-0.1, 1.4; p = 0.091) between V1 and V2 with CWP and no significant change from V2 to V3 (-0.3 [-0.9, 0.3]; p = 0.484).

A greater proportion of participants in CWP vs UC achieved MCID in AQLQ symptom domain (71% vs 31%; p = 0.024), AQLQ activity domain (53% vs 19%; p = 0.041) and AQLQ environmental domain (65% vs 19%; p = 0.008; Table 2, Figure 3). There was no between-group difference in the proportion achieving MCID in AQLQ emotional domain at one year (CWP 53%, UC 38%; p= 0.373).

*Other outcomes*

No between-group differences were detected across the three visits in MRC dyspnoea, Hospital Anxiety and Depressions scores, eosinophils, FeNO, or peak flow (e-Table 4). Change in post-bronchodilator FEV_1_ from baseline to one-year was greater with CWP (12.2%, 95% CI 2.1, 22.3) than UC (-2.8%, 95% CI –12.5, 6.8; p=0.020).

*Per protocol analysis*

Ten participants from the CWP group completed the one-year programme and attended V3 and were included for per protocol analysis compared to the 16 in UC. Mean difference in ACQ6 between groups was not significant over one year (CWP vs UC: -0.5, 95%CI –1.6, 0.6; p = 0.328), though repeated measures ANOVA suggested a trend towards improvement with CWP, F(2,18) = 3.023, p = 0.074, partial eta squared = 0.251 (e-Table 6). Post-hoc tests with Bonferroni correction in the CWP group showed a mean difference from V1-V2 of –0.81 (95%CI –1.68, 0.06; p = 0.069) and no change between V2-V3 (0.20, 95%CI –0.55, 0.95; p = 1.000). 6 (60%) of participants in CWP achieved MCID in ACQ6 compared to UC (4 [25%]; p = 0.109).

A greater proportion of participants achieved MCID improvement with CWP compared to UC at one year (e-Table 7) in overall AQLQ score (80% vs 6% respectively, p<0.001), AQLQ symptom domain (80% vs 31% respectively, p = 0.016), AQLQ activity domain (70% vs 19% respectively, p = 0.015) and AQLQ environmental domain (80% vs 19%, p = 0.004). No between-group difference was seen in AQLQ emotional domain.

Improvements in overall AQLQ score over one year with CWP (median change 0.8, IQR 0.5 to 1.8) were observed compared to UC (0.1, IQR –0.2 to 0.2; p = 0.003), and repeated measures ANOVA within the CWP group was significant, F(2,18) = 4.099, p = 0.034, partial eta squared = 0.313 (e-Table 6). Post-hoc tests with Bonferroni correction in the CWP group showed a mean difference from V1-V2 of 1.12 (95% CI 0.11, 2.13; p = 0.030) and no change between V2-V3 (-0.38, 95% CI –1.30, 0.54; p = 0.776). A similar result was observed in the AQLQ environmental domain with a mean between-group difference of 1.8 (95%CI 0.4, 3.2; p = 0.017) favouring CWP at one year. Repeated measures ANOVA confirmed a significant difference in the CWP group over one year, F(1.2,10.4) = 5.190 with Greenhouse-Geisser adjustment, p = 0.041, partial eta squared = 0.366. There was no between group difference in AQLQ symptom, activity or emotional domain scores, however, trends towards improvement with CWP in AQLQ symptom and activity domains were observed with repeated measures ANOVA (F[2,18] = 3.413, p = 0.055, partial eta squared = 0.275; F[2,18] = 2.747, p = 0.091, partial eta squared = 0.234 respectively). Post-hoc tests with Bonferroni correction for AQLQ symptom domain showed improvement with CWP from V1-V2 (mean change 1.23; 95%CI 0.24, 2.22; p = 0.016) with no difference between V2-V3 (-0.68; 95%CI –1.96, 0.60; p = 0.457). Post-hoc tests with Bonferroni correction for AQLQ activity domain showed a trend toward improvement with CWP from V1-V2 (0.78; 95%CI –0.08, 1.64; p = 0.080) with no difference from V2-V3 (-0.08; 95%CI –1.00, 0.84; p = 1.000). Repeated measures showed improvement for both CWP and UC in AQLQ emotional domain (F[2,18] = 4.814, p = 0.021, partial eta squared = 0.348; F[2,30] = 3.622, p = 0.039, partial eta squared = 0.195 respectively).

Median annualised number of oral corticosteroid courses reduced in the CWP group from 3 (IQR 2 to 5) at V1 to 0 (0 to 3) at V3 (Friedman chi-squared (2) = 7.9, p = 0.019) with no change observed in UC from 3 (2 to 5) at V1 to 2 (0 to 3) at V3 (Friedman chi-squared (2) = 0.4, p = 0.824; Table E6). No between-group difference was observed.

Median MRC dyspnoea scores improved in CWP from 3 (3 to 3) at V1 to 2 (2 to 3) at V3 (Friedman chi-squared (2) = 7.5, p = 0.024) with unchanged results observed in UC from 3 (3 to 4) at V1 to 3 (3 to 4) (Friedman chi-squared (2) = 0.4, p = 0.824).

Anthropomorphic measures were identical to those reported in the intention-to-treat analysis (e-Table 6).

**Supplementary tables**

**e-Table 1: Comparison of participants that attended one-year follow-up against those that did not**

|  | Did not attend (n = 4) | Did attend (n = 29) | p value |
| --- | --- | --- | --- |
| Age, yrs | 57.5 (49.9, 65.1) | 52.6 (47.9, 57.4) | 0.452 |
| Female sex | 4 (100.0) | 16 (55.2) | 0.136 |
| Smoking status:  Current smoker  Ex-smoker  Lifelong non-smoker | 0 (0.0)  1 (25.0)  3 (75.0) | 1 (3.4)  18 (62.1)  10 (34.5) | 0.295 |
| Age at asthma diagnosis, yrs | 31.8 (-3.7, 67.2) | 31.0 (23.2, 38.9) | 0.949 |
| Duration of asthma, yrs | 25.8 (-11.4, 62.9) | 21.7 (16.0, 27.3) | 0.630 |
| Atopy | 1 (25.0) | 23 (79.3) | 0.052 |
| Allergic rhinitis | 1 (25.0) | 16 (55.2) | 0.335 |
| Perennial rhinitis | 1 (25.0) | 14 (48.3) | 0.607 |
| Nasal polyps | 2 (50.0) | 2 (6.9) | 0.062 |
| Nasal surgery | 1 (25.0) | 2 (6.9) | 0.330 |
| Eczema | 1 (25.0) | 11 (37.9) | 1.000 |
| GORD | 3 (75.0) | 25 (86.2) | 0.500 |
| ILO/DFB | 1 (25.0) | 7 (24.1) | 1.000 |
| Psychological illness | 3 (75.0) | 14 (48.3) | 0.601 |
| Emphysema | 0 (0.0) | 5 (17.2) | 1.000 |
| Bronchiectasis | 0 (0.0) | 1 (3.4) | 1.000 |
| SAFS/ABPA | 0 (0.0) | 9 (31.0) | 0.555 |
| Diabetes mellitus | 1 (25.0) | 3 (10.3) | 0.431 |
| Hypertension | 2 (50.0) | 6 (20.6) | 0.241 |
| Cardiac disease | 1 (25.0) | 6 (20.6) | 1.000 |
| Osteopenia/osteoporosis | 2 (50.0) | 12 (41.4) | 1.000 |
| BDP equivalent dose, mcg* | 1600 (1600 to 1900) | 1600 (1600 to 2000) | 0.770 |
| Maintenance prednisolone | 1 (25.0) | 4 (13.9) | 0.500 |
| Prednisolone dose, mg* | 2 (NA) | 5 (2 to 9) | 1.000 |
| Azithromycin | 1 (25.0) | 6 (20.6) | 1.000 |
| Biologics | 1 (25.0) | 11 (37.9) | 1.000 |
| Previous 12 months*:  Prednisolone courses  OOH GP attendances  ED attendances  Hospital admissions  ICU admissions | 3 (2 to 4)  1 (0 to 4)  0 (0 to 0)  0 (0 to 0)  0 (0 to 0) | 3 (2 to 5)  0 (0 to 0)  0 (0 to 0)  0 (0 to 1)  0 (0 to 0) | 0.690  0.321  0.852  0.472  1.000 |
| Weight, kg* | 108.9 (99.0 to 131.6) | 101.5 (91.3 to 119.5) | 0.348 |
| BMI, kg/m^2^* | 44.9 (39.0 to 49.0) | 36.8 (33.9 to 41.8) | 0.082 |
| Waist circumference, cm | 129.9 (116.0, 143.7) | 120.3 (115.2, 125.4) | 0.177 |
| Hip circumference, cm | 134.3 (124.8, 143.8) | 121.5 (114.9, 128.0) | 0.156 |
| Waist-to-hip ratio* | 0.98 (0.93 to 0.99) | 1.02 (0.94 to 1.06) | 0.246 |
| Waist-to-height ratio | 0.81 (0.73, 0.90) | 0.73 (0.70, 0.77) | 0.075 |
| MRC dyspnoea score* | 4 (2 to 5) | 3 (3 to 4) | 0.690 |
| ACQ6 | 3.1 (1.5, 4.7) | 2.6 (2.2, 3.0) | 0.425 |
| AQLQ:  Overall  Symptom domain  Activity domain  Emotional domain  Environmental domain | 3.6 (2.7, 4.6)  3.3 (2.2, 4.3)  3.9 (2.9, 4.9)  3.3 (1.3, 5.3)  4.4 (3.7, 5.1) | 3.9 (3.5, 4.4)  3.9 (3.5, 4.4)  3.8 (3.3, 4.3)  3.9 (3.2, 4.5)  4.1 (3.6, 4.7) | 0.642  0.289  0.878  0.521  0.702 |
| HAD:  Anxiety score  Depression score | 12 (10, 14)  10 (2, 19) | 8 (7, 9)  8 (7, 10) | 0.069  0.452 |
| Eosinophils, x10^9/L* | 0.07 (0.02 to 0.19) | 0.11 (0.08 to 0.43) | 0.214 |
| FeNO, ppb* | 15 (NA) | 17 (10 to 29) | 0.808 |
| PEF, L/min | 286 (199, 372) | 386 (339, 432) | 0.121 |
| Spirometry, %:  Pre-BD FEV1  Pre-BD FEV1/FVC  Post-BD FEV1 | 60.8 (36.0, 85.5)  69.5 (54.5, 84.6)  65.8 (33.2, 98.3) | 72.4 (65.7, 79.0)  70.4 (66.8, 74.0)  74.3 (67.9, 80.7) | 0.217  0.865  0.362 |
| 6MWD, m | 208 (-88, 503) | 329 (292, 366) | **0.048** |
| Continuous variables described as mean (95% confidence intervals) or median (first quartile to third quartile), latter denoted by*.  Categorical variables described as no. (%).  P values show comparison of CWP vs UC using independent t test or Mann Whitney U test (latter denoted by *) for continuous and chi-squared or Fisher’s exact for categorical variables.  Abbreviations: ABPA (Allergic Bronchopulmonary Aspergillosis); ACQ6 (Asthma Control Questionnaire-6); AQLQ (Asthma Quality of Life Questionnaire); BD (Bronchodilator); BDP (Beclomethasone dipropionate); BMI (Body Mass Index); CWP (Counterweight Plus); DFB (Dysfunctional breathing); ED (Emergency Department); FeNO (Fractional exhaled Nitric Oxide); FEV_1_ (Forced Expiratory Volume in 1 second); FVC (Forced Vital Capacity); GORD (Gastro-oesophageal Reflux Disease); HAD (Hospital Anxiety and Depression scale); H2A (H2-receptor antagonists); ICU (Intensive Care Unit); ILO (Inducible Laryngeal Obstruction); LAMA (Long-acting anti-muscarinic); LPA (Low Physical Activity); MRC (Medical Research Council); MVPA (Moderate to Vigorous Physical Activity); OOH (Out-of-hours); PEF (Peak Expiratory Flow); ppb (parts per billion); PPI (Proton pump inhibitor); SAFS (Severe Asthma with Fungal Sensitisation); UC (Usual Care); 6MWD (6 minute Walk Distance) | | | |

**e-Table 2: Complete case intention-to-treat analysis of asthma control, quality of life and healthcare use variables across one year comparing CWP and UC**

|  | **Group** | **N** | **Mean (95% CI)/Median (IQR)** | | | **Repeated measures ANOVA/Friedman test** | | **V1-V3** | |
| --- | --- | --- | --- | --- | --- | --- | --- | --- | --- |
|  |  |  | V1 | V2 | V3 | p value | Effect size | Change in variable | P value |
| ACQ6 | CWP | 13 | 2.5 (1.9, 3.1) | 1.9 (1.1, 2.7) | 2.2 (1.2, 3.2) | 0.185 | 0.137 | -0.3 (-1.1, 0.5) | 0.639 |
|  | UC | 16 | 2.7 (2.2, 3.3) | 2.9 (2.3, 3.6) | 2.6 (2.0, 3.3) | 0.465 | 0.050 | -0.1 (-0.7, 0.6) |  |
| AQLQ | CWP | 13 | 4.0 (3.3, 4.7) | 4.9 (4.2, 5.6) | 4.5 (3.7, 5.3) | **0.040** | 0.235 | 0.7 (-0.3 to 1.3)** | 0.121 |
|  | UC | 16 | 3.8 (3.2, 4.5) | 3.9 (3.4, 4.5) | 3.9 (3.3, 4.6) | 0.914 | 0.006 | 0.1 (-0.2 to 0.2)** |  |
| AQLQ symptom | CWP | 13 | 4.0 (3.3, 4.7) | 5.1 (4.2, 5.9) | 4.5 (3.6, 5.4) | **0.040** | 0.236 | 0.6 (0.1 to 1.0)** | 0.249 |
|  | UC | 16 | 3.9 (3.2, 4.6) | 4.1 (3.6, 4.7) | 4.2 (3.5, 4.9) | 0.315 | 0.074 | 0.2 (-0.3 to 1.0)** |  |
| AQLQ activity | CWP | 13 | 4.0 (3.3, 4.7) | 4.5 (3.7, 5.4) | 4.3 (3.4, 5.2) | 0.256 | 0.107 | 0.3 (-0.6, 1.2) | 0.463 |
|  | UC | 16 | 3.7 (3.0, 4.3) | 3.5 (2.9, 4.2) | 3.6 (2.9, 4.3) | 0.916 | 0.006 | -0.1 (-0.7, 0.6) |  |
| AQLQ emotional | CWP | 13 | 3.8 (2.7, 5.0) | 5.6 (4.7, 6.4) | 4.5 (3.6, 5.4) | **0.003** | 0.392 | 0.6 (-0.5, 1.7) | 0.562 |
|  | UC | 16 | 3.9 (3.0, 4.8) | 4.6 (3.9, 5.2) | 4.3 (3.5, 5.0) | **0.039** | 0.195 | 0.3 (-0.2, 0.8) |  |
| AQLQ environmental | CWP | 13 | 4.0 (3.1, 4.8) | 4.7 (3.8, 5.7) | 4.8 (3.7, 5.8) | 0.152 | 0.145 | 0.8 (-0.2, 1.8) | 0.058 |
|  | UC | 16 | 4.2 (3.4, 5.0) | 3.7 (2.9, 4.5) | 3.7 (2.8, 4.6) | 0.432 | 0.054 | -0.5 (-1.5, 0.5) |  |
| Annualised Prednisolone courses* | CWP | 13 | 4 (2 to 6) | 0 (0 to 7) | 0 (0 to 2) | **0.005** | 0.412 | -3 (-5, -1) | 0.102 |
|  | UC | 16 | 3 (2 to 5) | 3 (0 to 6) | 2 (1 to 4) | 0.824 | 0.012 | -1 (-3, 1) |  |
| Variables described as mean (95%CI) and compared with repeated measures ANOVA (F-statistic and effect size η_p_^2^ [partial eta squared]). unless non-parametric (denoted by *): these variables described as median (IQR) and compared with Friedman chi-squared (effect size Kendall’s W).  V1-V3 variables described as mean (95%CI) and compared with independent t test, unless non-parametric (denoted by**) where variables described as median (IQR) and compared with Mann Whitney test.  Annualised variable compares change from baseline data (No. of events in prior 12 months) to 52 weeks ([No. of events × 365] / No. of d between visits).  Abbreviations: ACQ6 (Asthma Control Questionnaire 6), ANOVA (analysis of variance), AQLQ (Asthma Quality of Life Questionnaire), CWP (Counterweight-Plus weight management programme), UC (Usual Care), V1/V2/V3 (Visit 1/2/3), η_p_^2^ (partial eta squared). | | | | | | | | | |

**e-Table 3: Comparison of CWP participants across 16-weeks of those that attended one-year follow-up against those that did not**

|  | CWP Group | V1 | V2 | Mean difference (95% CI) | p value |
| --- | --- | --- | --- | --- | --- |
| Weight, kg | Did not attend V3 | 115.6 (63.4, 167.7) | 112.5 (8.3, 216.7) | -12.5 (-75.7, 50.7) | 0.242 |
|  | Did attend V3 | 105.7 (97.7, 113.6) | 93.0 (83.4, 102.6) | -13.7 (-18.3, -9.1) | **<0.001** |
| ACQ6 | Did not attend V3 | 3.3 (0.5, 6.1) | 2.7 (0.8, 4.6) | -0.6 (-3.8, 2.6) | 0.501 |
|  | Did attend V3 | 2.5 (1.9, 3.1) | 2.1 (1.3, 2.9) | -0.4 (-1.1, 0.2) | 0.193 |
| AQLQ | Did not attend V3 | 3.8 (2.6, 5.1) | 4.0 (3.0, 4.9) | 0.1 (-0.7, 1.0) | 0.578 |
|  | Did attend V3 | 3.9 (3.3, 4.6) | 4.9 (4.2, 5.6) | 1.0 (0.3, 1.6) | **0.006** |
| Variables described as mean (95%CI) and compared using independent t test  Abbreviations: ACQ6 (Asthma Control Questionnaire 6), AQLQ (Asthma Quality of Life Questionnaire), CWP (Counterweight-Plus weight management programme), V1/V2 (Visit 1/2) | | | | | |

**e-Table 4: Comparison of CWP participants at one-year by type 2 inflammatory status**

|  | Group | N | V1 | V3 | Change V1-V3 | p value |
| --- | --- | --- | --- | --- | --- | --- |
| ACQ6 | T2 High | 11 | 2.9 (2.2, 3.6) | 2.1 (1.2, 3.1) | -0.7 (-1.5, 0.1) | **0.067** |
|  | T2 Low | 6 | 2.2 (1.3, 3.1) | 2.3 (0.6, 3.9) | 0.1 (-1.3, 1.5) | 0.907 |
| AQLQ | T2 High | 11 | 3.7 (3.0, 4.4) | 4.4 (3.5, 5.3) | 0.7 (0.1, 1.3) | **0.036** |
|  | T2 Low | 6 | 4.3 (3.1, 5.5) | 4.6 (3.3, 5.9) | 0.3 (-1.6, 2.3) | 0.680 |
| AQLQ symptom | T2 High | 11 | 3.8 (3.0, 4.5) | 4.6 (3.7, 5.4) | 0.8 (0.2, 1.4) | **0.012** |
|  | T2 Low | 6 | 4.0 (2.7, 5.3) | 4.2 (2.8, 5.7) | 0.2 (-2.2, 2.6) | 0.833 |
| AQLQ activity | T2 High | 11 | 3.7 (3.0, 4.4) | 4.1 (3.1, 5.1) | 0.4 (-0.4, 1.2) | 0.303 |
|  | T2 Low | 6 | 4.4 (3.4, 5.5) | 4.7 (3.5, 5.9) | 0.3 (-1.4, 2.0) | 0.714 |
| AQLQ emotional | T2 High | 11 | 3.4 (2.3, 2.5) | 4.5 (3.5, 5.5) | 1.1 (0.3, 1.9) | **0.010** |
|  | T2 Low | 6 | 4.3 (2.3, 6.4) | 4.5 (3.6, 5.5) | 0.2 (-2.4, 2.7) | 0.862 |
| AQLQ environmental | T2 High | 11 | 3.7 (2.9, 4.6) | 4.6 (3.5, 5.8) | 0.9 (-0.1, 1.9) | **0.081** |
|  | T2 Low | 6 | 4.7 (3.7, 5.7) | 5.0 (3.8, 6.2) | 0.3 (-1.2, 1.7) | 0.656 |
| Annualised Prednisolone courses | T2 High | 11 | 4 (2 to 6) | 0 (0 to 2) | -2 (-6 to 0) | **0.003** |
|  | T2 Low | 6 | 4 (2 to 4) | 0 (0 to 2) | -4 (-4 to 0) | 0.340 |
| MRC dyspnoea | T2 High | 9 | 3 (3, 4) | 3 (2, 4) | 0 (-1, 1) | 0.438 |
|  | T2 Low | 4 | 3 (2, 4) | 3 (0, 5) | -1 (-4, 3) | 0.703 |
| HADS Anxiety | T2 High | 9 | 9 (6, 11) | 8 (4, 12) | 0 (-3, 3) | 0.876 |
|  | T2 Low | 4 | 9 (6, 12) | 7 (5, 10) | 0 (-2, 2) | 1.000 |
| HADS Depression | T2 High | 9 | 7 (5, 10) | 8 (4, 12) | 0 (-3, 4) | 0.779 |
|  | T2 Low | 4 | 8 (3, 13) | 6 (-2, 14) | 0 (-11, 10) | 0.945 |
| Continuous variables described as mean (95% confidence intervals) or median (first quartile to third quartile), latter denoted by*.  P value compares V1 vs V3 using paired t test or if non-parametric (denoted by*) Wilcoxon signed-rank test.  Annualised variable compares change from baseline data (No. of events in prior 12 months) to 52 weeks ([No. of events × 365] / No. of d between visits).  Abbreviations: ACQ6 (Asthma Control Questionnaire 6), AQLQ (Asthma Quality of Life Questionnaire), ED (Emergency Department), HADS (Hospital Anxiety and Depression Score), ICU (Intensive Care Unit), MRC (Medical Research Council), OOH (Out-Of-Hours), T2 (Type 2), V1/V3 (Visit 1/3). | | | | | | |

**e-Table5: Comparison of CWP participants at one-year of those that lost ≥10% total body weight against those that lost <10% body weight**

|  | Group | N | V1 | V3 | Change V1-V3 | p value |
| --- | --- | --- | --- | --- | --- | --- |
| ACQ6 | <10% weight loss | 3 | 2.7 (-1.0, 6.3) | 3.1 (-0.7, 6.9) | 0.5 (-4.6, 5.6) | 0.732 |
|  | ≥10% weight loss | 7 | 2.1 (1.3, 3.0) | 1.1 (0.2, 1.9) | -1.1 (-1.9, -0.3) | **0.018** |
| AQLQ | <10% weight loss | 3 | 3.8 (-1.4, 9.1) | 3.4 (1.9, 4.9) | -0.4 (-6.7, 5.9) | 0.809 |
|  | ≥10% weight loss | 7 | 4.3 (3.4, 5.2) | 5.5 (4.7, 6.3) | 1.2 (0.4, 2.1) | **0.011** |
| AQLQ symptom | <10% weight loss | 3 | 4.5 (-0.3, 9.3) | 3.2 (-0.7, 7.1) | -1.3 (-7.7, 5.1) | 0.483 |
|  | ≥10% weight loss | 7 | 4.1 (3.1, 5.1) | 5.4 (4.4, 6.4) | 1.3 (0.4, 2.2) | **0.010** |
| AQLQ activity | <10% weight loss | 3 | 3.2 (-1.7, 8.2) | 3.2 (2.5, 3.9) | 0.0 (-5.5, 5.5) | 0.982 |
|  | ≥10% weight loss | 7 | 4.4 (3.6, 5.2) | 5.4 (4.4, 6.5) | 1.0 (0.0, 2.0) | 0.052 |
| AQLQ emotional | <10% weight loss | 3 | 4.3 (-1.9, 10.5) | 3.7 (2.4, 4.9) | -0.7 (-7.4, 6.1) | 0.713 |
|  | ≥10% weight loss | 7 | 4.3 (2.6, 6.0) | 5.6 (4.6, 6.5) | 1.3 (-0.2, 2.8) | 0.074 |
| AQLQ environmental | <10% weight loss | 3 | 3.4 (-3.4, 10.1) | 4.4 (2.0, 6.9) | 1.1 (-4.8, 7.0) | 0.519 |
|  | ≥10% weight loss | 7 | 4.5 (3.7, 5.3) | 5.8 (4.8, 6.9) | 1.3 (0.4, 2.2) | 0**.011** |
| Annualised Prednisolone courses | <10% weight loss | 3 | 2 (2 to 3) | 3 (2 to 5) | 1 (-9, 10) | 1.000 |
|  | ≥10% weight loss | 7 | 3 (3 to 5) | 0 (0 to 0) | -4 (-7, -1) | **0.018** |
| MRC dyspnoea | <10% weight loss | 3 | 3 (3 to 4) | 3 (3 to 4) | 1 (-5, 6) | 0.655 |
|  | ≥10% weight loss | 7 | 3 (3 to 3) | 2 (1 to 2) | -1 (-2, -1) | **0.024** |
| Continuous variables described as mean (95% confidence intervals) or median (first quartile to third quartile), latter denoted by*.  P value compares V1 vs V3 using paired t test or if non-parametric (denoted by*) Wilcoxon signed-rank test.  Annualised variable compares change from baseline data (No. of events in prior 12 months) to 52 weeks ([No. of events × 365] / No. of d between visits).  Abbreviations: ACQ6 (Asthma Control Questionnaire 6), ANOVA (analysis of variance), AQLQ (Asthma Quality of Life Questionnaire), V1/V3 (Visit 1/3). | | | | | | |

**e-Table 6: Per protocol analysis comparing asthma control and quality of life over one year between CWP and UC**

|  | **Group** | **N** | **Mean (95% CI)/Median (IQR)** | | | **Repeated measures ANOVA/Friedman test** | | **V1-V3** | |
| --- | --- | --- | --- | --- | --- | --- | --- | --- | --- |
|  |  |  | V1 | V2 | V3 | p value | Effect size | Change in variable | P value |
| ACQ6 | CWP | 10 | 2.3 (1.6, 3.0) | 1.5 (0.7, 2.3) | 1.7 (0.9, 2.5) | 0.074 | 0.251 | -0.6 (-1.6, 0.4) | 0.328 |
|  | UC | 16 | 2.7 (2.2, 3.3) | 2.9 (2.3, 3.6) | 2.6 (2.0, 3.3) | 0.465 | 0.050 | -0.1 (-0.7, 0.6) |  |
| AQLQ | CWP | 10 | 4.2 (3.3, 5.0) | 5.3 (4.6, 5.9) | 4.9 (4.1, 5.7) | **0.034** | 0.313 | 0.8 (0.5 to 1.8) | **0.003** |
|  | UC | 16 | 3.8 (3.2, 4.5) | 3.9 (3.4, 4.5) | 3.9 (3.3, 4.6) | 0.914 | 0.006 | 0.1 (-0.2 to 0.2) |  |
| AQLQ symptom | CWP | 10 | 4.2 (3.3, 5.1) | 5.4 (4.7, 6.2) | 4.8 (3.9, 5.6) | 0.055 | 0.275 | 0.8 (0.3 to 1.3) | 0.201 |
|  | UC | 16 | 3.9 (3.2, 4.6) | 4.1 (3.6, 4.7) | 4.2 (3.5, 4.9) | 0.315 | 0.074 | 0.2 (-0.3 to 1.0) |  |
| AQLQ activity | CWP | 10 | 4.1 (3.2, 4.9) | 4.8 (4.0, 5.6) | 4.8 (3.9, 5.6) | 0.091 | 0.234 | 0.7 (-0.4, 1.8) | 0.174 |
|  | UC | 16 | 3.7 (3.0, 4.3) | 3.5 (2.9, 4.2) | 3.6 (2.9, 4.3) | 0.916 | 0.006 | -0.1 (-0.7, 0.6) |  |
| AQLQ emotional | CWP | 10 | 4.3 (3.2, 5.4) | 6.0 (5.2, 6.9) | 5.0 (4.1, 5.9) | **0.021** | 0.348 | 0.7 (-0.8, 2.2) | 0.594 |
|  | UC | 16 | 3.9 (3.0, 4.8) | 4.6 (3.9, 5.2) | 4.3 (3.5, 5.0) | **0.039** | 0.195 | 0.3 (-0.2, 0.8) |  |
| AQLQ environmental | CWP | 10 | 4.2 (3.2, 5.2) | 5.1 (4.1, 6.1) | 5.4 (4.2, 6.6) | **0.041** | 0.366 | 1.2 (0.3, 2.2) | **0.017** |
|  | UC | 16 | 4.2 (3.4, 5.0) | 3.7 (2.9, 4.5) | 3.7 (2.8, 4.6) | 0.432 | 0.054 | -0.5 (-1.5, 0.5) |  |
| Weight, kg* | CWP | 9 | 101.7 (95.5 to 112.0) | 88.8 (82.0 to 90.7) | 87.1 (85.9 to 93.3) | **<0.001** | 0.778 | -14.0 (-14.8 to -9.2)** | **0.015** |
|  | UC | 8 | 106.0 (80.9 to 128.0) | 105.6 (80.9 to 124.9) | 108.6 (87.1 to 145.5) | 0.417 | 0.109 | 1.9 (-7.3 to 7.9)** |  |
| BMI, kg/m^2^* | CWP | 9 | 37.5 (35.6 to 41.8) | 32.6 (30.1 to 35.1) | 33.1 (31.4 to 37.6) | **0.004** | 0.613 | -4.2 (-6.4, -2.0) | **0.036** |
|  | UC | 8 | 37.1 (31.5 to 47.8) | 37.2 (31.0 to 47.0) | 37.5 (32.6 to 54.4) | 0.417 | 0.109 | -0.1 (-3.6, 3.4) |  |
| Annualised Prednisolone courses* | CWP | 10 | 3 (2 to 5) | 2 (0 to 8) | 0 (0 to 3) | **0.019** | 0.397 | -2 (-5, 0) | 0.314 |
|  | UC | 16 | 3 (2 to 5) | 3 (0 to 6) | 2 (1 to 4) | 0.824 | 0.012 | -1 (-3, 1) |  |
| Parametric variables described as mean (95%CI) and compared with repeated measures ANOVA (effect size η_p_^2^) and non-parametric (denoted by *) described as median (IQR) and compared with Friedman chi-squared (effect size Kendall’s W).  V1-V3 variables described as mean (95%CI) and compared with independent t test, unless non-parametric (denoted by**) where variables described as median (IQR) and compared with Mann Whitney test.  Annualised health-care use variables compare change from baseline data (No. of events in prior 12 months) to 52 weeks ([No. of events × 365] / No. of d between visits).  Abbreviations: ACQ6 (Asthma Control Questionnaire 6), ANOVA (analysis of variance), AQLQ (Asthma Quality of Life Questionnaire), CWP (Counterweight-Plus weight management programme), UC (Usual Care), V1/V2/V3 (Visit 1/2/3), η_p_^2^ (partial eta squared). | | | | | | | | | |

**e-Table 7: Proportion of per protocol participants achieving MCID in asthma control and quality of life scores at 16-weeks and 52-weeks**

|  | **16-weeks** | | | **52-weeks** | | |
| --- | --- | --- | --- | --- | --- | --- |
|  | **CWP (n=10)** | **UC (n=16)** | **p value** | **CWP (n=10)** | **UC (n=16)** | **p value** |
| ACQ6 | 7 (70.0) | 3 (18.8) | **0.015** | 6 (60.0) | 4 (25.0) | 0.109 |
| AQLQ | 7 (70.0) | 5 (31.3) | 0.105 | 8 (80.0) | 1 (6.3) | **<0.001** |
| AQLQ Symptoms | 8 (80.0) | 7 (43.8) | 0.109 | 8 (80.0) | 5 (31.3) | **0.016** |
| AQLQ Activity | 6 (60.0) | 4 (25.0) | 0.109 | 7 (70.0) | 3 (18.8) | **0.015** |
| AQLQ Emotional | 7 (70.0) | 12 (75.0) | 1.000 | 4 (40.0) | 6 (37.5) | 1.000 |
| AQLQ Environmental | 6 (60.0) | 5 (31.3) | 0.228 | 8 (80.0) | 3 (18.8) | **0.004** |
| P value compares CWP vs UC using either chi-squared or Fisher’s exact test.  Abbreviations: ACQ6 (Asthma Control Questionnaire 6), AQLQ (Asthma Quality of Life Questionnaire), CWP (Counterweight-Plus weight management programme), UC (Usual Care) | | | | | | |
